# Single-Cell Transcriptomic Profiling of GL261 Glioblastoma Cells Reveals Gene Expression Signatures Underlying Tumorigenicity

**DOI:** 10.1007/s10571-025-01635-0

**Published:** 2025-12-16

**Authors:** Colton E. Troxel, Ruby A. Olvera, Emily A. Freko, Suely S. C. Soeiro, Thandiswa T. Mdluli, Richard L. Daniels

**Affiliations:** Department of Biology, The College of Idaho, Idaho Caldwell, USA

**Keywords:** GL261, Glioblastoma, scRNA-Seq, P2RX7, MMP, Tumor microenvironment

## Abstract

Glioblastoma is a lethal primary brain tumor with poor prognosis. Tumor cells exhibit substantial phenotypic variation, complicating treatment. As functional diversity is driven by underlying transcriptional states, characterizing tumor cell gene expression is essential for understanding tumor biology and therapeutic response. The GL261 tumor cell line is a common pre-clinical model system for investigating glioblastoma pathobiology. However, global gene expression patterns in this model are unknown. Here we describe the use of single-cell RNA sequencing (scRNA-Seq) to investigate transcriptional profiles of 5764 adherent and 4951 neurosphere GL261 cells, generating 133,442,221 sequenced reads. Following Principal Component Analysis (PCA) for dimensionality reduction, we applied Uniform Manifold Approximation and Projection (UMAP) to visualize transcriptionally distinct subpopulations (clusters) of GL261 cells grown adherently or as neurospheres. Highly expressed and differentially expressed genes were identified. Because the neurosphere phenotype is known to be more tumorigenic, we further examined differentially expressed genes with gene ontology expression analysis. We found that upregulated genes in neurosphere cells are associated with angiogenesis, cell adhesion, and cell signaling pathways. In addition, we specifically examined gene expression patterns of matrix metalloproteinases and purinergic receptors, glioblastoma drug targets known to be important for promoting tumor infiltration into adjacent healthy tissue. We found that P2RX7, MMP15 and MMP16 are upregulated in neurosphere cells, indicating a potential role for these genes in tumor formation. Together these results reveal global transcriptional profiles of GL261 cells, establish a resource for further scRNA-Seq-based analyses, and give insight into gene expression changes relevant to glioblastoma tumor development.

## Introduction

Glioblastoma Multiforme (GBM) is a lethal primary astrocytoma characterized by rapid proliferation and a high degree of invasiveness; median survival is 15 months. The age-adjusted incidence of GBM is 4 per 100,000, with more than 12,000 people diagnosed annually in the United States (Tamimi and Juweid 2017; Grochans et al. 2022; Alifieris and Trafalis 2015). Treatment consists of maximal surgical resection, radiation, and chemotherapeutic regimens with alkylating agents such as temozolomide or carmustin (Alifieris and Trafalis 2015).

Within solid tumors, including GBM, tumor cells exhibit wide transcriptional diversity, leading to the tumor being composed of subpopulations of phenotypically distinct cells. Intra-tumor cell heterogeneity is a consistently-identified feature of solid tumors and increased variation is associated with worse patient outcomes in GBM, squamous cell carcinomas, and cancers of the prostate, breast, and colon (Haffner et al. 2021; Chan and Buczacki 2021; Mroz et al. 2013; Becker et al. 2021; Lüönd et al. 2021; Guan et al. 2018). These differences underlie significant therapeutic challenges, in part via a selective mechanism where subpopulations of tumor cells develop resistance to anti-tumor drug compounds and therefore survive to proliferate (Dagogo-Jack and Shaw 2018). Differences in patient outcomes and disease progression may also be explained by the impacts of heterogenous tumor cells on tumor pathophysiology. For example, GBM cells derived from human tumor biopsies show phenotypic differences in adhesive properties, migratory speed, and immune escape phenotypes (Manini et al. 2020; Rezk et al. 2020).

The study of gene expression in tumors has been enhanced by single-cell RNA Sequencing (scRNA-Seq), a technique that enables high-resolution gene expression analyses at the level of individual tumor cells (Li et al. 2021; González-Silva et al. 2020; Zhang et al. 2021). Several projects have specifically characterized human GBM tumors using scRNA-Seq, and these efforts have been summarized in a recent review (Ordóñez-Rubiano et al. 2025). Besides examining GBM tumor cell heterogeneity, reports have compared transcriptomic profiles of infiltrating GBM cells with other cells in the tumor and investigated gene expression differences in GBM tumor cells before and after temozolomide-based treatment (Patel et al. 2014; Darmanis et al. 2017; Wang et al. 2022).

While most studies of single-cell transcriptomic profiles have examined human tumors, comparably few have examined gene expression in the most commonly used cell-based model systems of GBM which underlie pre-clinical drug trials and fundamental investigations of tumor pathobiology. One study used single-cell transcript profiling to examine global gene expression in U-87 cells (Wang et al. 2019), and two studies have investigated CT-2 A and GL261-derived tumor cells following engrafting of these cell lines into mice (Mikolajewicz et al. 2024; García-Vicente et al. 2025). Though investigations of in-vivo gene expression in human tumors and engrafted animal tumors reveal individual tumor attributes and molecular signatures common to both human tumors and animal model tumors, many pre-clinical studies rely solely on in-vitro cell-based model systems for experimentation on fundamental aspects of tumor cell physiology, motility, and drug responses (Gómez-Oliva et al. 2021; Slika et al. 2023; Tavener et al. 2021). Thus there remains a significant gap in the understanding of global gene expression in the in-vitro model systems most commonly used to study GBM.

One of the most widely-used model system to study GBM is the murine derived GL261 cell line (Szatmári et al. 2006; Slika et al. 2023). These cells can be cultured as adherent cells (GL261-AC) or as free-floating cell aggregates called neurospheres (GL261-NS). We and others have shown that phenotypic changes occur when GL261-AC cells are induced to form neurospheres by supplementing the culture media with growth factors (Akbasak et al. 1991; Pellegatta et al. 2006; Szatmári et al. 2006; Strong and Daniels 2017). Notably, neurospheres more closely resemble the in-vivo tumor phenotype in terms of molecular profiles, more readily form tumors when engrafted into syngeneic mice, and have higher rates of lethality (Pellegatta et al. 2006).

Two gene families that have been widely studied in human tumors, animal models, and cell-based systems due to their therapeutic potential are matrix metalloproteinases (MMPs) and purinergic receptors (P2RX and P2RY). MMPs play a role in extracellular matrix degradation and their abnormal expression is thought to lead to a tumor’s ability to infiltrate nearby healthy tissue. This role for MMPs in tumor pathophysiology also makes them a potential therapeutic target, and high levels of MMP2, MMP9, MMP15, MMP16 have been shown to be expressed in GBM tumor cells and associated with poor prognoses (Pullen et al. 2018; Marino et al. 2023; Han et al. 2021; Aitchison et al. 2025; Thome et al. 2020). Purinergic signaling in the tumor microenvironment makes ATP receptors another promising drug target (Matyśniak et al. 2022; Bergamin et al. 2019; Vultaggio-Poma et al. 2020). In particular, the ionotropic ATP receptor P2RX7 is known to be associated with GBM tumor formation, and we and others have shown that P2RX7 is expressed and functional in both GL261-AC and GL261-NS cells (Tamajusuku et al. 2010; Strong and Daniels 2017; Strong et al. 2018).

This project seeks to characterize global gene expression in GL261-AC and GL261-NS tumor cells using scRNA-Seq. We will specifically examine differential gene expression that occurs with the transcriptional changes associated with the tumorigenic GL261-NS phenotype. We hypothesize that compared to adherent cells, gene expression in neurosphere cells more closely approximates gene expression characteristics of tumors. We will additionally characterize expression and differential expression of P2RX and P2RY ATP receptors and MMPs. Together, these studies will reveal fundamental gene expression profiles of the widely used GL261 model system and identify gene expression patterns associated with GBM tumorigenesis.

## Methods

### Cell Culture of Adherent and Neurosphere GL261 Cells

GL261 cells were provided by the NCI-Frederick Cancer Research Tumor Repository (Frederick, MD). Cells were cultured adherently in DMEM (Gibco/Thermo Fisher Scientific) with 10% FBS (R&D Systems) and 1% penicillin-streptomycin (Millipore Sigma) at 37.0 °C in 5.0% CO₂ using 75 cm cell culture-treated flasks (Corning/Fisher Scientific). Cells were passaged by trypsinization at 80–90% confluency every 2–4 days. To induce neurosphere formation prior to single-cell RNA sequencing, media was supplemented with 20 ng/mL of Epidermal Growth Factor (Peprotech/Thermo Fisher Scientific), 20 ng/mL Fibroblast Growth Factor (Peprotech/Thermo Fisher Scientific), and 1:50 B-27 (Gibco/Thermo Fisher Scientific) to the cell culture media. Neurosphere culture media was replaced every 3–5 days by pelleting cells via centrifugation and re-suspending in new culture media with continued growth factor and B-27 supplementation. Neurospheres were cultured 12 days prior to shipment for scRNA-Seq analysis.

### Single Cell RNA Sequencing and Seurat-based Data Analysis

Sample preparation: Adherent and neurosphere cells were prepared according to instructions from Azenta Life Sciences. Adherent cells were grown to ~ 80% confluency and collected from culture flasks by trypsinization. Neurospheres were collected by centrifugation and triturated using a series of progressively smaller pipets tips to break up clusters into single cells (5 ml serological pipette, 1 ml pipette tip, 200 µl pipette tip, and finally a fire-polished glass Pasteur pipette). For both adherent and neurosphere cells, cell concentrations were determined with a hemacytometer and cell viability was determined using trypan blue staining. Samples were placed in freezing media (culture media with 10% DMSO) and 1 ml aliquots were transferred to cryotubes, placed in a -20 °C freezer for 1 h, then in a -80 °C freezer overnight. Samples were shipped on dry ice overnight to Azenta Life Sciences.

Sample processing, sequencing, and read quantification: Library construction and sequencing of viable cells was performed by Azenta Life Sciences using the 10x Genomics single cell 3’ v3 protocol and Illumina NovaSeq X Plus sequencing platform. Preliminary data processing and mapping reads to transcripts and cells (including sample demultiplexing, barcode processing, and unique molecular identifier counting) was performed using the Cellranger-7.0.1 data pipeline. A total of 5,764 adherent cells and 4,951 neurosphere cells were sequenced.

Quality control and Data Analysis: Data were processed and analyzed using the RStudio IDE (2024.04.1 + 748) with R (4.5.0) with the Seurat R package (5.1.0) (Satija et al. 2015; Hao et al. 2024). Mitochondrial genes (13) and ribosomal genes (101) were removed from further analysis. We also removed low quality cells, including adherent cells containing fewer than 1000 total RNA transcripts (nCount_RNA < 1000) and fewer than 1250 unique RNA transcripts (nFeature_RNA < 1250). For neurosphere cells, thresholds were set at 1000 total RNA transcripts and 2250 unique RNA transcripts. Log-based normalization and scaling was performed using Seurat’s NormalizeData and ScaleData functions with default parameters.

Dimensionality Reduction and Clustering: Principal components (PCs) of the dataset were found using the RunPCA function on the top 2000 highly variable genes identified using the FindVariableFeatures function. We determined the first 17 PCs would be used for adherent cell clustering, and the first 18 for neurosphere cell clustering. Using the FindNeighbors function, appropriate PCs were defined in each data set. Visualization of cells on a 2-dimensional projection was performed using Uniform Manifold Approximation and Projection (UMAP) with the RunUMAP function with default parameters (1.0 resolution), with clusters defined based on shared nearest neighbor (SNN) graph analysis. We used adherent cells as the reference sample for anchor identification while performing the integration of adherent and neurosphere cell datasets. The FindIntegrationAnchors function allowed for the determination of cells that shared the mutual nearest neighbor characteristics prior to data integration with the IntegrateData function.

Batch effects correction and gene expression visualization: Differentially expressed genes in the individual and integrated datasets were identified using the Findallmarkers function. Bubble plots, heat maps, and UMAP heat maps were created with the DotPlot, DoHeatmap and FeaturePlot functions, respectively. Batch correction was performed when integrating the adherent and neurosphere datasets using Canonical Correlation Analysis (CCA) through Seurat’s IntegrateData function.

Gene Ontology Expression Analysis: Neurosphere cell genes expressed with a log_2_-fold change greater than 1 compared to adherent cells were identified and used for gene ontology expression analyses. Analyses were performed using the Panther Classification System (PANTHER) (The Gene Ontology Consortium et al. 2023; Thomas et al. 2003, 2022).

### Live Cell Calcium Imaging (Calcium microfluorimetry)

Glass 8-well chamber slides (Thermoscientific Nunc Lab-Tek) were coated for 30–120 min with poly-L-lysine (Sigma-Aldrich) by adding 200 µL of Poly-L-Lysine to each well and incubating at room temperature. Adherent GL261 cells were trypsinized, plated in the 8-well chamber slides at a density of 5 × 10^4^ cells/mL, and allowed to incubate for 24 h. Prior to imaging, growth media was removed and the cells were washed twice with 200 µL of calcium imaging buffer (CIB; 130 mM NaCl, 3.6 mM KCl, 1.8 mM CaCl2, 1.0 mM MgCl2, 10 mM D-glucose, 10 mM HEPES, pH 7.4 adjusted with HCl and NaOH). After the final wash, 200 µL of 5 µM fura-2 (Invitrogen) in CIB was placed in each well, and the chamber slide was placed in the dark for 30 min at room temperature. After incubation, wells were washed twice with 200 µL CIB. Test solutions containing BzATP in CIB were delivered by pipet while imaging. Ratiometric pseudocolored images were captured with a Nikon Eclipse Ti-S epifluorescent inverted microscope equipped with a dual 340/380 nm excitation filter wheel (Sutter) and associated Nikon Digital Sight DS-U3 camera. Images and videos were analyzed using the Nikon Elements software package.

### RNA Isolation and PCR for P2RX7 Transcript Variant Detection

Total RNA from GL261 cells was extracted using a Qiagen RNeasy kit. QIA Shredder columns were used for cell lysis (Qiagen). Reverse transcription of mRNA transcripts to first-strand cDNA was performed using a Qiagen Omniscript kit and oligo-dT primers. Isoform-specific primers (Integrated DNA Technologies) were used in PCR (AllTaq PCR core kit, Qiagen) and amplicons were detected via gel electrophoresis and compared against a Gene Ruler 100 bp Plus ladder (Thermo Scientific). Amplicon identity was confirmed by gel extraction (Qiagen QIAquick Gel Extraction Kit) and sequencing (Azenta Life Sciences). To design primers and perform analyses, consensus coding sequences (CCDS) of the 5 known Mus musculus P2RX7 isoforms were obtained from the National Center for Biotechnology Information (NCBI): isoform A (CCDS19652.1), isoform B (CCDS19651.1), isoform C (CCDS 51644.1), isoform D (CCDS19653.1), isoform E (CCDS89983.1). These correspond directly to the known transcript variants 1, 2, 3, 4 and 5, respectively for P2RX7, but without any untranslated sequences. Forward, reverse, and sequencing primers were designed using the NCBI BLAST tool.

## Results

**Fig. 1:**
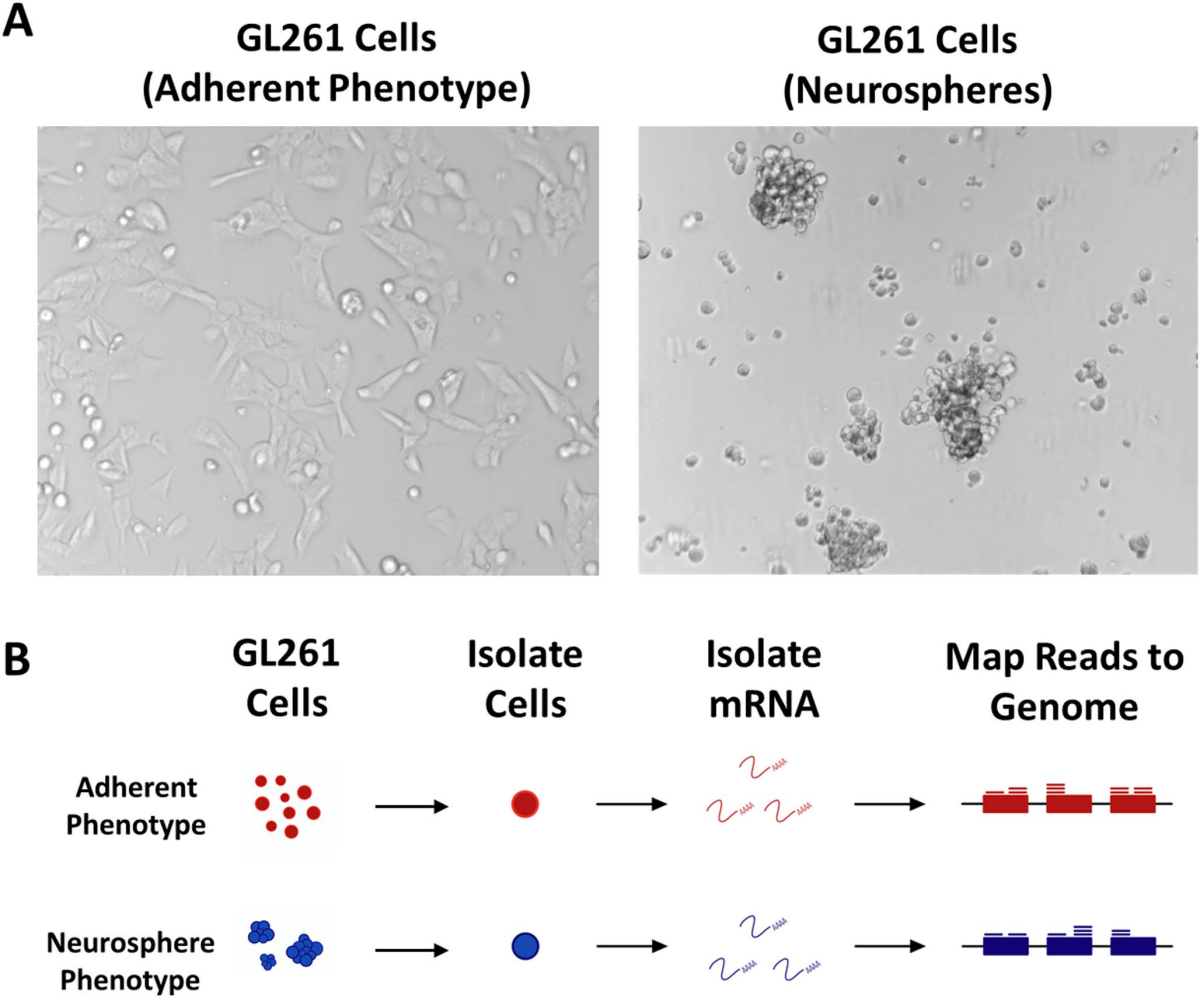
Single-cell RNA-Sequencing of GL261 cells cultured adherently or as neurospheres. (A) Bright field images (200X magnification) of GL261 cells cultured adherently (left) or as free-floating cell aggregates called neurospheres (right). The neurosphere phenotype is induced when cell culture media is supplemented with growth factors. (B) Flow chart showing a brief overview of single-cell RNA Sequencing (scRNA-Seq). GL261 cells were isolated and mRNA was extracted, reverse transcribed, and sequenced. Transcripts (represented by small lines in the figure) were mapped to known genes (blocks) in the genome and quantified for each cell

Adherent and neurosphere GL261 cells were cultured and prepared for single-cell RNA sequencing (Fig. 1). To better understand gene expression and tumor cell heterogeneity, we examined gene expression at single cell resolution in both the adherent and neurosphere GL261 cell phenotypes. In total, we generated scRNA-Seq profiles of 5,764 adherent cells and 4951 neurosphere cells. Among the adherent cells we obtained 133,442,221 total sequenced reads (mean detection rate of 23,151 reads per cell) and 94.2% of these were mapped to the mouse genome. Figure 1B graphically depicts the isolation of total mRNA and subsequent mapping of sequenced mRNA transcript fragments to known genes.

**Fig. 2:**
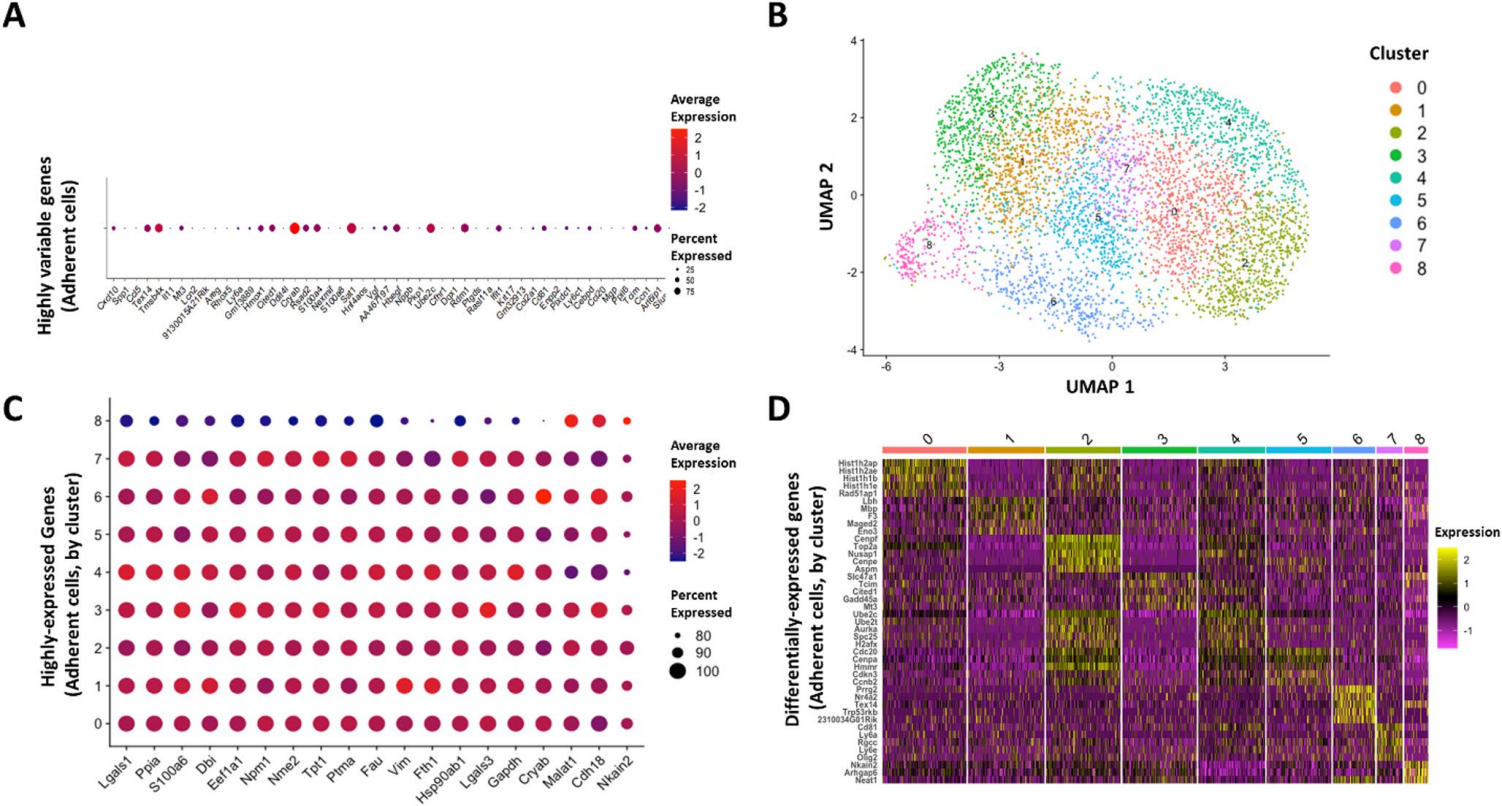
Adherent cell gene expression analysis, UMAP clustering and visualization. (A) Top 50 most highly variable genes expressed in adherent GL261 cells, ordered from left to right. Expression levels were log normalized within each cell to the total transcript count. Dot color represents the average log normalized expression across all adherent cells, and dot size represents the percentage of cells that express the gene. (B) UMAP cluster plot of adherent cells. Dots represent individual cells, and cells in close proximity share similar gene expression profiles. Dot colors represent unique clusters. (C) Genes observed to have the highest average (log normalized) expression in each cluster. (D) Heat map showing the top differentially expressed genes in each cluster relative to other clusters. Yellow blocks indicate higher differential expression

A total of 21,597 genes were represented across all adherent cells, with a median gene expression of 3,002 genes detected per cell. Among all neurosphere cells we obtained 163,315,478 total sequenced reads (mean detection rate of 32,986 reads per cell) with 95.9% mapped to the mouse genome. A total of 22,555 genes were represented across all neurosphere cells, with median gene expression of 4,178 genes per cell. The complete dataset is available at the National Center for Biotechnology Information (NCBI) Gene Expression Omnibus (GEO) database, with Accession # GSE277850. Quality control, data processing, and analyses were performed as described in the methods using the R-based Seurat scRNA-Seq analysis package. We began with quality control and data processing. Briefly, cells with low total gene expression were removed from the dataset. We also removed sequencing reads corresponding to mitochondrial or ribosomal genes, as these are disproportionately expressed in all cell types (Subramanian et al. 2022). Following this, 5,073 adherent cells and 4,300 neurosphere cells remained. Gene expression levels were then log normalized so as to allow cell-to-cell comparisons. Our analysis began with the identification of the most highly variable genes expressed in adherent GL261 cells (relative to average expression). The top 50 most highly variable genes are depicted in a bubble plot which shows both order of magnitude of expression relative to the mean (color) and the prevalence of expression as a percentage of total cells in the adherent population (size of bubble) (Fig. 2A). For example, we observed that the genes Cryab, Sat1, and Ube2c were expressed at levels 2–3 orders of magnitude (100-1000X) greater than mean values, and their expression was detected in greater than 75% of cells. To address the question of whether gene expression is largely consistent across the adherent cell population or whether adherent cells are heterogeneous in their expression profiles, we visualized cell-by-cell gene expression patterns using UMAP clustering (Fig. 2B). Briefly, UMAP analysis begins with the assumption that genes and the magnitude of their expression constitute a multi-dimensional dataset. PCA-based dimensionality reduction was performed on the most highly variable genes, which results in the identification of a smaller subset of genes (principal components) that most contribute to the variance in the dataset. Lastly, UMAP projects cells into a two-dimensional space based on their principal component values, while a shared nearest neighbor graph is used independently to define clusters of cells with similar expression profiles. Our UMAP plot shows 9 Seurat-identified clusters (subpopulations of adherent cells). Following UMAP visualization, we further investigated the gene expression characteristics unique to each cluster. We identified the top 10 most highly-expressed genes in each cluster and visualized with a dot plot. If each cluster was entirely unique in gene expression, 90 genes would be shown. Our dot plot shows 19 unique genes, indicating overlap between the top 10 highly-expressed genes in each cluster (Fig. 2C). As in 2A, dot size represents the percentage of cells expressing the gene, and dot color represents log-normalized expression. Finally, we constructed a heat map of the genes that are characteristic of each cluster; that is, genes that have the highest differential expression (yellow blocks) in each cluster relative to other clusters (Fig. 2D). Color indicates z-scored log-normalized expression.

**Fig. 3:**
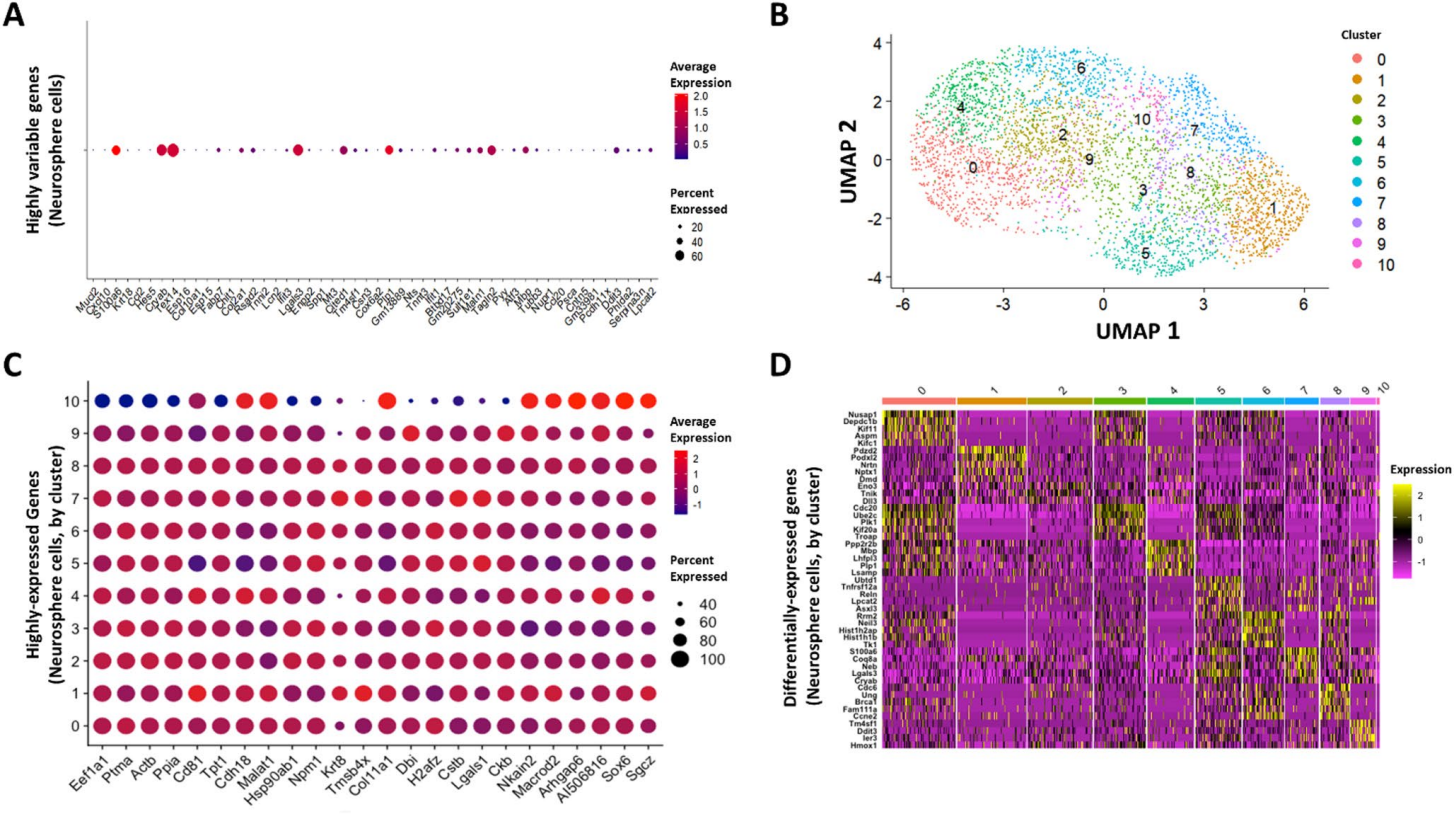
Neurosphere cell gene expression analysis, UMAP clustering and visualization. (A) Top 50 most highly variable genes expressed in neurosphere GL261 cells, ordered from left to right. Expression levels were log normalized within each cell to the total transcript count. Dot color represents the average log normalized expression across all neurosphere cells, and dot size represents the percentage of cells that express the gene. (B) UMAP cluster plot of neurosphere cells. Dots represent individual cells, and cells in close proximity share similar gene expression profiles. Dot colors represent unique clusters. (C) Genes observed to have the highest average (log normalized) expression in each cluster. (D) Heat map showing the top differentially expressed genes in each cluster relative to other clusters. Yellow blocks indicate higher differential expression

Following these investigations of gene expression in adherent cells, we repeated the same analyses in neurosphere cells (Fig. 3). Notably, the top 50 most variable genes in the neurosphere cells are largely distinct from the adherent cells, with only 14 in common between the two phenotypes (Cxcl10, Spp1, Tex14, Mt3, Lcn2, Gm13889, Cited1, Cryab, Rsad2, Ccl2, Enpp2, Ifit1, Col2a1, Ccl20) (Fig. 3A). UMAP clustering was again used to visualize subpopulations of neurosphere cells with broadly similar gene expression profiles, and 11 clusters were identified (Fig. 3B). The top 10 highly-expressed genes expressed in each cluster were visualized as a bubble plot (Fig. 3C). If each cluster was entirely unique in gene expression, 110 genes would be identified. Our dot plot shows 24 unique genes, indicating overlap between the top 10 highly-expressed genes in each cluster. In total, 11 genes (of 32 total) were highly expressed in at least one cluster of adherent or neurosphere cells: Lgals1, Ppia, Dbi, Eef1a1, Npm1, Tpt1, Ptma, Hsp90ab1, and Malat1, Cdh18, and Nkain2. As in 3A, dot size represents the percentage of cells expressing the gene, and dot color represents log-normalized expression. We constructed a heat map of the neurosphere genes that are characteristic of each cluster; that is, genes that have the highest differential expression (yellow blocks) in each cluster relative to other clusters (Fig. 3D). Color indicates z-scored log-normalized expression.

**Fig. 4:**
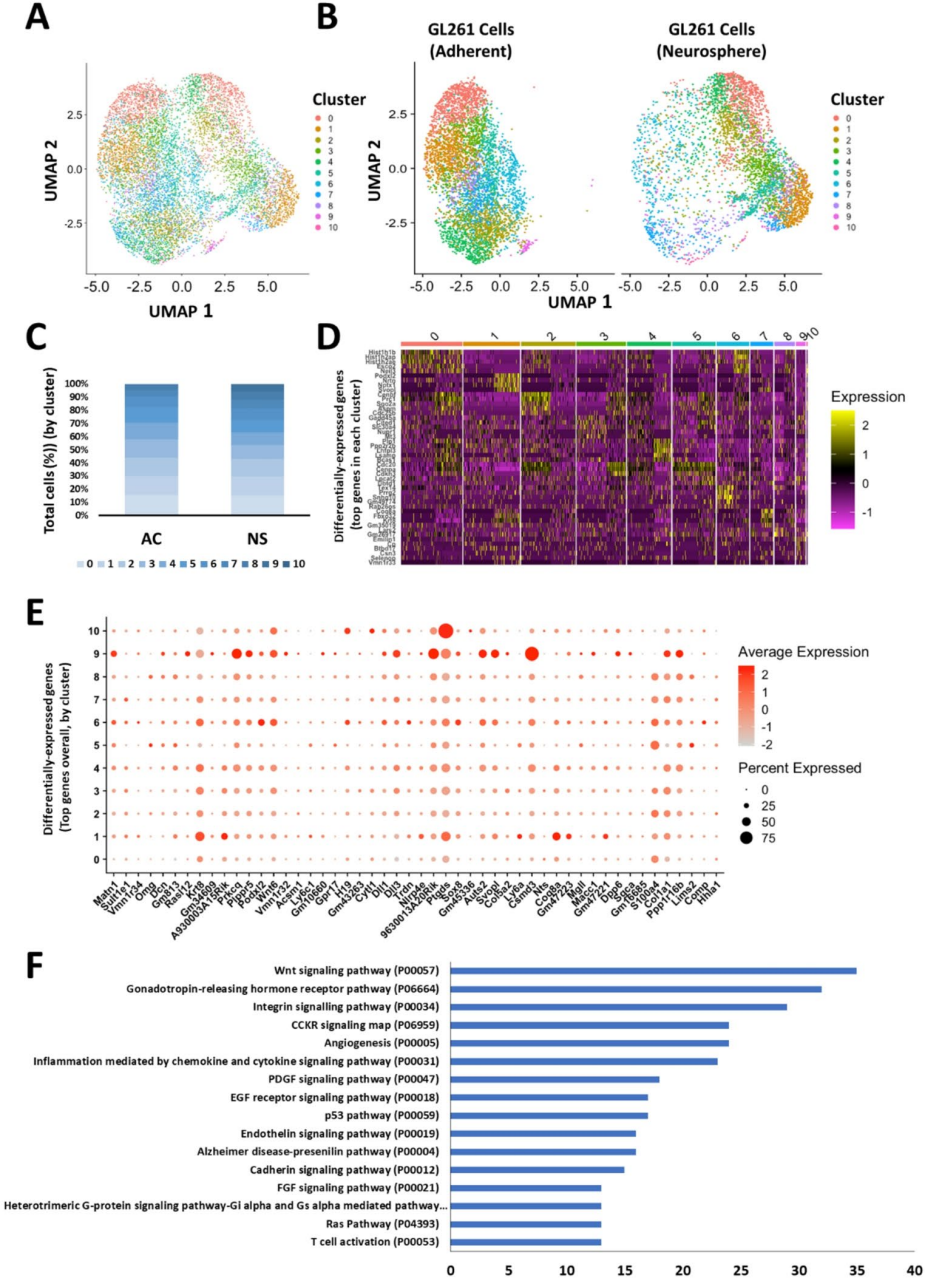
Visualizing gene expression difference in adherent and neurosphere GL261 cells. (A) UMAP cluster plot of integrated adherent and neurosphere GL261 cell scRNA-Seq datasets. Dots represent individual cells, and cells in close proximity share similar gene expression profiles. Dot colors represent unique clusters. (B) Identical data as presented in A, but with cells separated by phenotype. (C) Stacked bar chart showing the percentage of total cells in each cluster for the adherent and neurosphere phenotypes. (D) Heat map showing the top differentially expressed genes in each cluster relative to other clusters. Yellow blocks indicate higher differential expression. (E) Genes with the greatest differential expression between the adherent and neurosphere phenotypes, shown for each cluster. (F) Gene ontology expression analysis of upregulated genes in the neurosphere phenotype compared to the adherent cell phenotype. The indicated pathways are represented by least 12 upregulated genes

Next, we sought to visualize subpopulations (clusters) of GL261 cells after integrating the adherent and neurosphere datasets to determine the extent to which gene expression profiles of subpopulations of cells varied with phenotype (Fig. 4). After correcting for batch effects, we created UMAP cluster plots that displayed both datasets together on one projection (Fig. 4A) and extracted by phenotype (Fig. 4B). We observed that neurosphere cells were not as uniformly concentrated in their associations with each other as adherent cells. One example of this is that neurosphere cells in cluster 7 were observed in close proximity to many cells in other clusters, indicating heterogeneity in gene expression not present in the same adherent cell cluster. To determine whether the induction of the neurosphere phenotype corresponded to over-representation of cells in one or more clusters, we calculated the percentage of cells in each cluster for each phenotype and represented this with a stacked bar chart (Fig. 4C). We found that adherent cells and neurosphere cells were both represented in approximately the same proportions in clusters 0–8 (proportions of adherent cells in each of these clusters ranged from 0.45 to 0.60). However, two clusters in the integrated dataset (clusters 9 and 10) were composed solely of neurosphere cells (230 cells in cluster 9 and 24 cells in cluster 10). Note that cluster 10 contains so few cells as to not be visible in the figure. To better understand the unique gene expression of each cluster, a heatmap was constructed of this integrated dataset (Fig. 4D). As in the previous heat maps, genes shown are those that have the highest differential expression (yellow blocks) in each cluster relative to other clusters. Color indicates z-scored log-normalized expression. We also examined different expression of genes without respect to clusters, and generated a list of the overall top upregulated genes among neurosphere cells. The top 50 most upregulated genes across all neurosphere cells are shown plotted by cluster on a bubble plot, where dot size represents the percentage of cells expressing the gene, and dot color represents log-normalized expression (Fig. 4E). One of the primary goals of this study is to understand the underlying reasons why neurospheres more readily form tumors as compared to adherent cells. To interpret the functional significance of differentially expressed genes, we performed a gene ontology (GO) term enrichment analysis using the PANTHER (Protein ANalysis THrough Evolutionary Relationships) database. When compared to adherent cells, neurosphere cells contained 1664 genes upregulated at least 2-fold over the mean log-normalized expression level in each cell. Of these, PANTHER mapped 745 genes to 107 pathways represented by 1–32 genes each. Figure 4F depicts pathways represented by more than 12 genes per pathway. These results show that the neurosphere phenotype is associated with known pathways including second-messenger signaling (Wnt, gonadotropin-releasing hormone receptor, EGF, FGF, PDGF, Ras), cell adhesion (integrin, cadherin), and angiogenesis. Top differentially-expressed genes in each cluster were also investigated using PANTHER to perform GO term enrichment analyses; the same top pathways emerged when analyses were performed by cluster as for the entire dataset (data not shown).

**Fig. 5:**
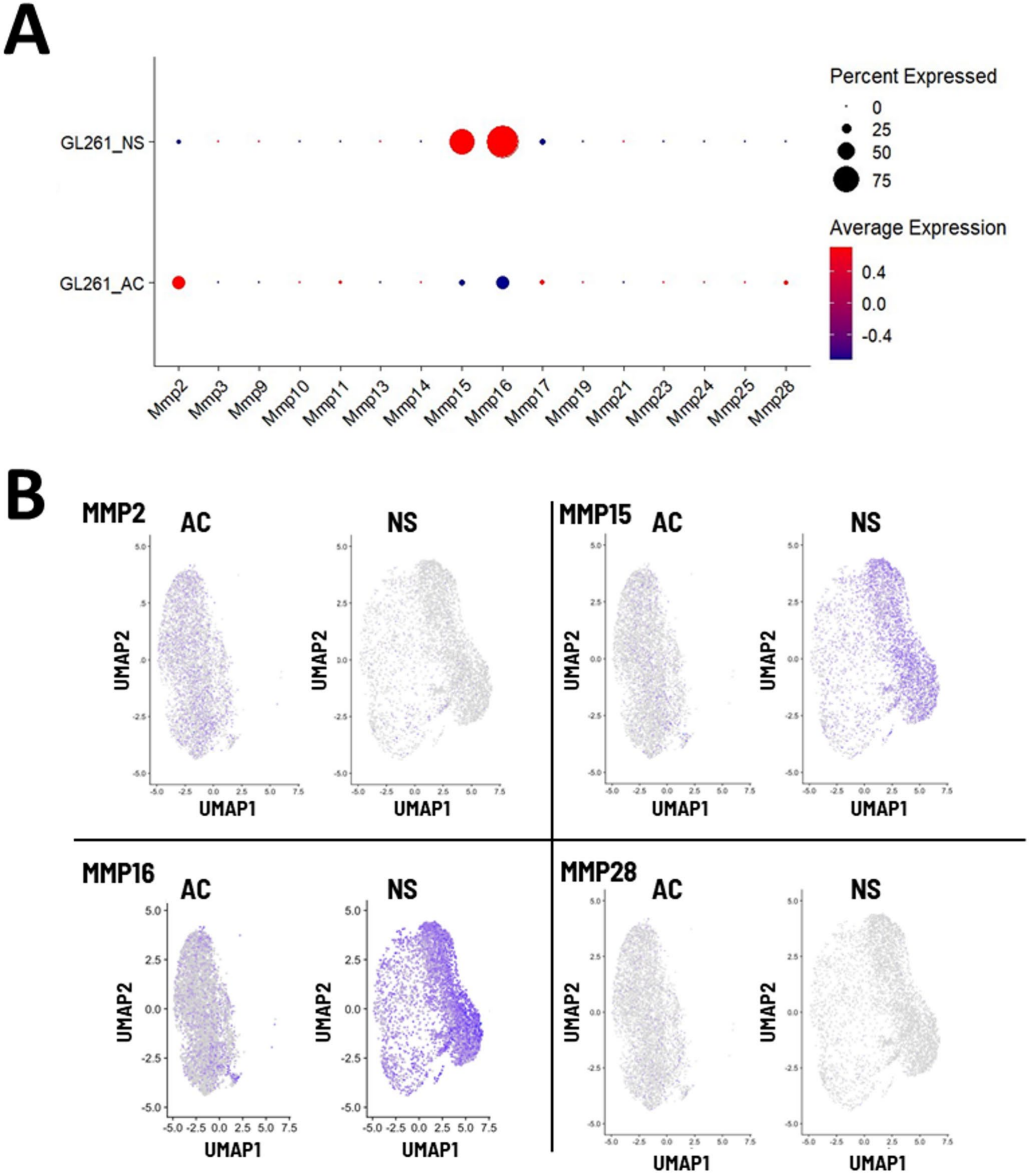
Visualizing gene expression of matrix metalloproteinases (MMPs) in GL261 cells. (A) Bubble plot showing overall gene expression of MMP in adherent or neurosphere GL261 cells. Dot color represents the average log-normalized expression across all cells in each category, and dot size represents the percentage of cells that express the gene. (B) UMAP plots showing cell-specific expression of indicated MMP genes (indicated by blue)

**Fig. 6:**
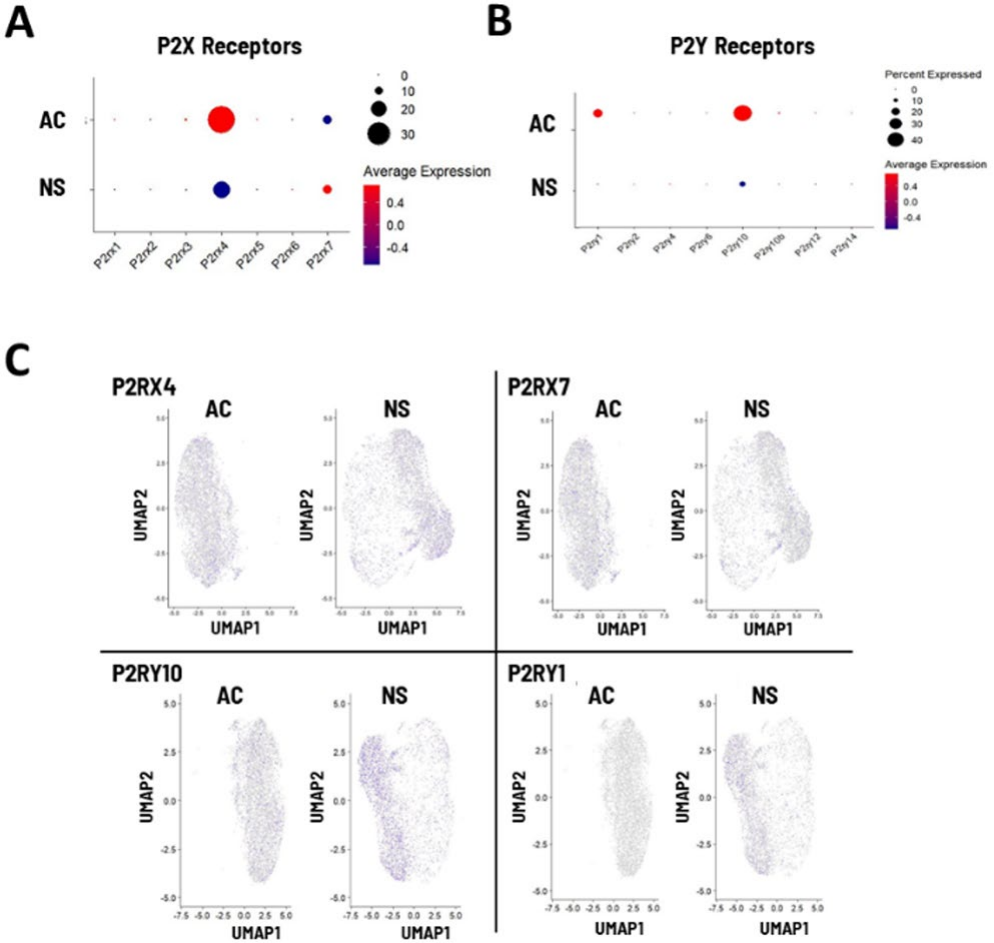
Visualizing gene expression of purinergic P2X and P2Y receptors in GL261 cells. (A) Bubble plot showing overall gene expression of P2X receptors in adherent or neurosphere GL261 cells. Dot color represents the average log-normalized expression across all cells in each category, and dot size represents the percentage of cells that express the gene. (B) Bubble plot showing overall gene expression of P2Y receptors in adherent or neurosphere GL261 cells. (C) UMAP plots showing cell-specific expression of indicated P2X and P2Y genes (indicated by blue)

We next tested the hypothesis that specific genes known to be involved in glioblastoma tumor formation are upregulated in the neurosphere phenotype. Matrix metalloproteinase (MMP) gene expression was visualized using a bubble plot (Fig. 5A). We found that in neurosphere cells, MMP15 and MMP16 were upregulated and each expressed in more than 50% of cells. This expression was also visualized in a cell-specific manner using a heat map overlaid on a UMAP plot (Fig. 5B). MMP2 and MMP28, which show no differences in expression between phenotypes, are visualized for visual comparison. Similarly, we visualized purinergic receptor expression, including the P2X and P2Y ATP receptors, using a bubble plot (Fig. 6A). We found that in neurosphere cells, P2RX7 was upregulated relative to adherent cells, while P2RX4 was downregulated. P2YR1 and 2YR10 gene expression was detected in adherent cells, but downregulated in the neurosphere phenotype. Cell-specific expression was again visualized with a heat map overlaid on UMAP plots for each gene (Fig. 6B).

**Fig. 7:**
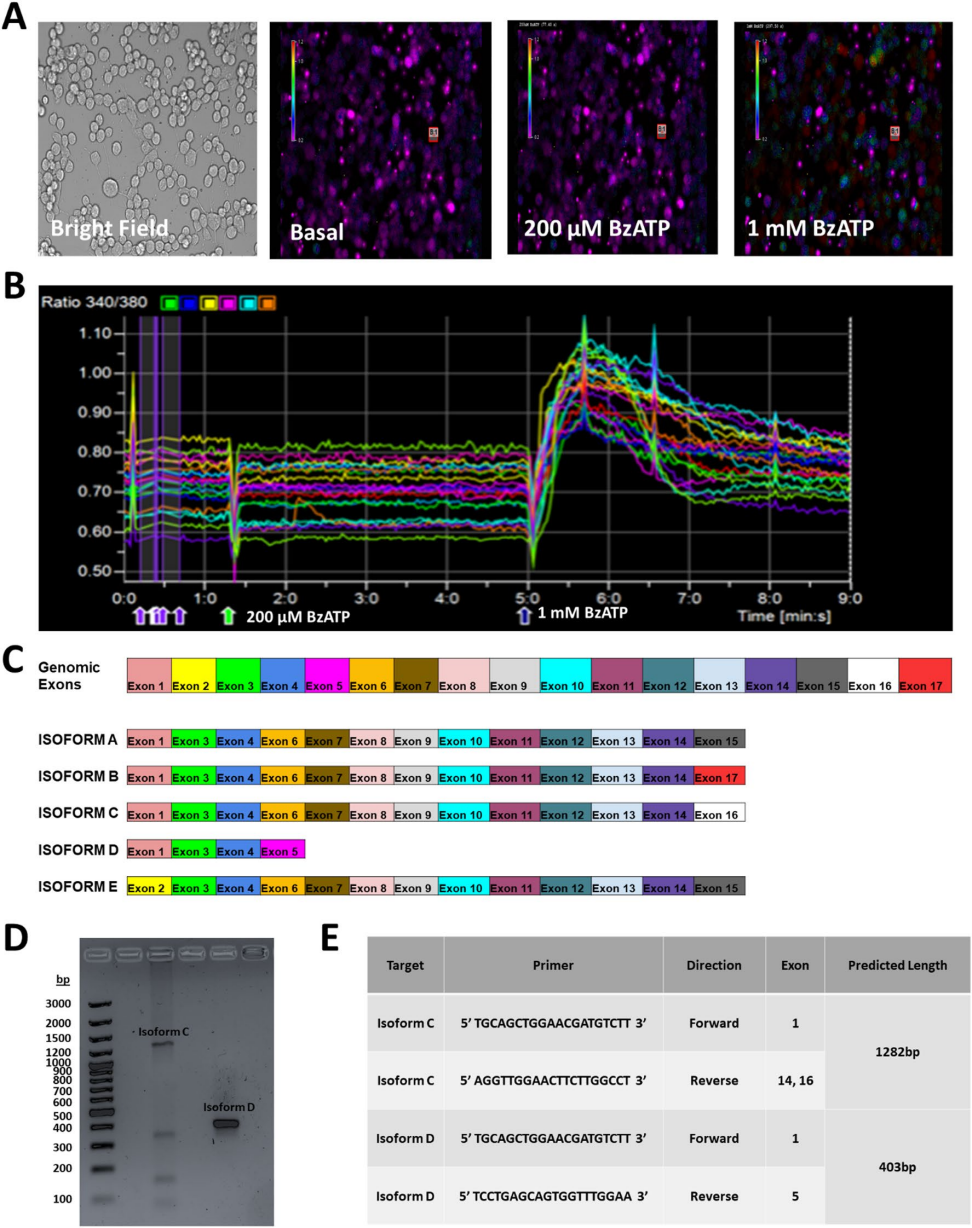
Calcium imaging of functional ATP responses in GL261 cells. (A) Bright field image of adherent GL261 cells at 200X magnification (left), followed by pseudocolored images representing cellular responses under basal conditions and after exposure to BzATP at the indicated concentrations. Warm colors represent increased intracellular calcium levels. (B) Graph illustrating representative traces of intracellular calcium response of 20 cells. Arrows on the x-axis indicate the time of BzATP application. (C) Chart showing order of all exons in genomic P2RX7 (top) followed by exons expressed in P2RX7 mRNA isoforms C and D. (D) DNA gel electrophoresis of PCR products obtained from primer combinations specific for isoforms C and D. (E) Primer sequences along with their target exons and predicted PCR product lengths.

Lastly, we sought to establish whether transcript expression data for the ionotropic ATP receptor P2RX7 corresponded to functional protein expression in GL261 cells. To test this we used live cell calcium imaging (calcium microfluorimetry) to observe intracellular calcium responses to BzATP, a P2RX7-specific agonist (Fig. 7). Pseudocolored images show an intracellular rise immediately following BzATP treatment, indicating that the channel was expressed and functional (Fig. 7A and B). We found that 20.8% of cells responded to treatments of 500 µM (*n* = 7), corresponding approximately to the percentage of cells identified using scRNA-Seq that express P2RX7 (Fig. 6). In mice, five isoforms of P2RX7 are known to be expressed in mouse tissue (isoforms A, B, C, D, and E). To determine which of these, if any is expressed and functional in GL261 cells, we isolated total cellular mRNA, reversed transcribed to cDNA, and used PCR to amplify transcripts that correspond to P2RX7 isoform consensus coding sequences (CCDS). Figure 4C shows all genomic exons and expressed exons that code for known P2RX7 isoforms. Using PCR with primer pairs specific for each CCDS, we found evidence of P2RX7 RNA transcripts that correspond to P2RX7 isoforms C and D. PCR products identified by gel electrophoresis were identified that matched expected values of 1282 bp and 403 bp, respectively (Fig. 7D and E). Following DNA gel electrophoresis, PCR products were sequenced to confirm isoform identity (data not shown). Expression of isoforms A, B and E was not detected.

## Discussion

This study characterized cell-specific gene expression in both adherent and neurosphere GL261 cells, a commonly used model system for studying glioblastoma tumor drug responses, motility, and immune interactions. We found that the most highly expressed genes in adherent and neurosphere cells were mostly different, with only 14 of the top 50 genes common to both phenotypes (Figs. 2 and 3). We also found that the sets of top differentially expressed genes in each UMAP cluster were mostly unique in adherent vs. neurosphere cells. Following data integration, we found that neurosphere cells contained a wider degree of transcriptional variation, as seen in the UMAP clusters in Fig. 4A and B. This finding is also supported by the straightforward initial scRNA-Seq characterization of transcript quantities prior to data analysis—more unique transcripts were detected in neurosphere cells (4178) than adherent cells (3002). UMAP clusters for both adherent and neurosphere cells were composed of approximately the same numbers of cells, suggesting that cells remain transcriptionally heterogenous and evenly dispersed into clusters in both the adherent and neurosphere phenotypes, rather than the alternative of observing cells primarily consolidated into fewer clusters (Fig. 4C).

A notable finding of our study was that GO term enrichment analysis of the top differentially expressed genes between neurosphere and adherent cells showed that neurosphere-specific upregulated genes were associated with pathways known to be important in tumorigenesis, including angiogenesis, cell attachment, and second messenger signaling pathways (Fig. 4F). These findings support our initial hypothesis that the neurosphere phenotype more closely approximates the tumor phenotype than adherent cells.

Following our studies of broad patterns of gene expression, we investigated gene expression specifically in families of matrix metalloproteases (MMPs) and purinergic receptors (P2RX and P2RY). We found that gene expression of MMP15 and MMP16 were upregulated in neurosphere cells, and the number of cells expressing MMP15 and MMP16 greatly increased (Fig. 5). Increased expression of MMP-family proteins is found in GBM tumors and associated with worse prognoses (Pullen et al. 2018; Marino et al. 2023; Han et al. 2021; Aitchison et al. 2025; Thome et al. 2020). Expression of MMP15 and MMP16 may contribute to the neurosphere cells’ ability to establish a tumor when engrafted into mice.

We also carried out more detailed analyses of purinergic receptors, as P2RX7 is considered a drug target for GBM. Previous studies from our lab have examined functional responses of GL261 cells (both adherent and neurosphere) to ATP, and have found that slightly fewer cells respond to ATP in the neurosphere phenotype (Strong et al. 2018; Strong and Daniels 2017). This study extends our previous work by examining the relative expression of specific ATP receptors and receptor subtypes. For example, we found that while adherent GL261 cells express a variety of receptors (especially P2RX4, P2RX7, P2RY1 and P2RY10), only P2RX7 was upregulated in neurosphere cells. An interesting related finding is that while the percentage of cells expressing P2RX7 remained similar, the percentage of cells expressing P2RX4, P2RY1 and P2RY10 decreased (Fig. 6). This aligns with previous work from our lab, which showed that fewer cells respond to ATP in neurosphere cells than adherent cells (Strong and Daniels 2017). We note a relevant caveat here regarding scRNA-Seq; it is not a method that is optimized for detecting transcripts present in very low quantities (Chen et al. 2019). Our findings do not rule out the expression of other channels in GL261 cells at low levels, and in fact other studies indicate GL261 adherent cells express other P2RX and P2RY transcripts as detected by PCR (Tamajusuku et al. 2010). Still, our results show that P2RX7 is a more significant part of ATP signaling in neurosphere cells compared to adherent cells.

We further investigated the role of P2RX7 using calcium imaging and pharmacological activation of the channel to screen for functional protein expression. We found that functional responses to BzATP (20.8% of cells, Fig. 7) approximately corresponded to scRNA-Seq-determined gene expression, which show P2RX7 transcripts expressed in 10–20% of cells (Fig. 6). Further investigation using RT-PCR showed that only P2RX7 isoforms C and D are expressed, indicating that these (alone or in combination) are likely responsible for detecting ATP. It is important to note that these isoforms have large structural differences, with isoform D transcripts containing only 4 exons, making it about one-third of the total length of the other isoform (and missing the entire C-terminus). This finding underscores the significance of understanding isoform-specific receptor-ligand interactions for future drug trials in this model system or in-vivo. Together, our results support a previously hypothesized role for P2RX7 in glioma tumorigenicity where truncated P2RX7 isoforms in the ATP-enriched tumor microenvironment are tonically active without leading to cytotoxic pore formation. This leads to a calcium-dependent increase in exocytosis of Vascular Endothelial Growth Factor (VEGF) and subsequent angiogenesis (Zanoni et al. 2022; Pegoraro et al. 2024; Di Virgilio et al. 2018; Adinolfi et al. 2023; Amoroso et al. 2015).

Though the GL261 cell line is a valuable platform for investigating tumor biology and pathobiology, there are important considerations as to the extent it recapitulates in-vivo tumor gene expression and phenotype. First, repeated passaging leads to selection of cells that thrive in culture, and thus cultured cells may differ in significant ways from those that thrive in the in-vivo tumor environment. Even though adherent and neurosphere cells were analyzed after approximately the same number of passages, the possibility exists that observed gene expression differences could arise from artificial selection of GL261 subclones due to rapid adaptation to neurosphere culture media. Furthermore, long-term culturing of GL261 cells has surely led to genomic and epigenetic modifications that diverge from the in-vivo tumor. Second, mouse-derived tumor models differ from human in-vivo tumors in significant ways, both in their in-vivo characteristics when engrafted into animal hosts and their gene expression profiles (Candolfi et al. 2007; Jacobs et al. 2011; Patrizii et al. 2018). The dataset described here contributes to the on-going effort to refine our understanding of murine model systems, and it would be valuable for future analyses to include this dataset (publicly available at the NCBI Gene Expression Omnibus database, GEO #GSE277850) in a broader effort to investigate species-specific differences in gene expression and to map the gene expression characteristics of the described cell clusters onto known astrocyte and human glioma subtypes.

Together, these results contribute to our understanding of gene expression in the GL261 model system and highlight the importance of continued investigation of matrix metalloproteinases and purinergic receptors as drug targets for GBM treatment.

## Data Availability

The datasets generated during and/or analyzed during the current study are available at the National Center for Biotechnology Information (NCBI) Gene Expression Omnibus (GEO) database, with Accession # GSE277850. https://www.ncbi.nlm.nih.gov/geo/query/acc.cgi? acc=GSE277850.
